# Morel-Lavallée Lesion in a Patient With Metformin-Induced B12 Deficiency: A Rare Complication Following a Fall

**DOI:** 10.7759/cureus.80480

**Published:** 2025-03-12

**Authors:** Vyshnavi Rallapalle, Yuyuan Wu, William Curry

**Affiliations:** 1 Heersink School of Medicine, University of Alabama at Birmingham, Birmingham, USA; 2 Birmingham VA Medical Center, University of Alabama at Birmingham, Birmingham, USA

**Keywords:** lower limb wounds, metformin therapy, morel-lavallée lesion (mll), types 2 diabetes, vit b12 deficiency

## Abstract

A Morel-Lavallée lesion (MLL) is a type of closed injury resulting in the internal shearing of superficial skin and subcutaneous tissue from the underlying fascial layer, usually occurring after traumatic accidents such as sports injuries or motor vehicle accidents. In this case, a 68-year-old man developed an MLL as a complication following a moderate-impact fall due to metformin-induced vitamin B12 deficiency. A known complication of metformin use is vitamin B12 deficiency, but this is often underdiagnosed due to a lack of routine screening. This case illustrates the importance of being aware of this complication and the many roles of vitamin B12 in terms of its effects on balance, proprioception, blood vessel integrity, and wound healing, emphasizing the importance of routine vitamin B12 screening in metformin users.

## Introduction

A Morel-Lavallée lesion (MLL) is a type of closed injury resulting in the internal shearing of superficial skin and subcutaneous tissue from the underlying fascial layer, which leads to fluid accumulation between the subcutaneous tissue and fascia [1]. MLLs often occur after traumatic events such as sports injuries or motor vehicle accidents [1]. However, they are frequently misdiagnosed as hematomas, fat necrosis, or soft tissue sarcomas, resulting in delayed diagnosis and management [1].

In this case, an MLL developed as a complication following a fall in a patient who had been using metformin over an extended period, leading to a vitamin B12 deficiency. Metformin is one of the most widely prescribed medications for the treatment of type 2 diabetes. However, a known side effect of metformin is that it interferes with vitamin B12 absorption in the ileum [2]. Chronic B12 deficiency can lead to macrocytic anemia, neuropathy, ataxia, psychiatric changes, and impaired wound healing, all of which can significantly impact diabetes patients [2,3]. The neuropathy and ataxia associated with B12 deficiency can lead to serious injuries from falls, especially in elderly patients. Despite the well-established association between metformin and B12 deficiency, this condition is often underdiagnosed due to the lack of routine screening of B12 levels in clinical practice.

We present the first reported case of an MLL occurring in association with vitamin B12 deficiency secondary to prolonged metformin use. To prevent complications associated with B12 deficiency in patients undergoing long-term metformin treatment, early B12 level screening and appropriate management with B12 supplementation are essential.

## Case presentation

Our case involves a 68-year-old male patient with a complex past medical history including hypertension, coronary artery disease requiring angioplasty, and insulin-dependent diabetes mellitus. His medication history is notable for the use of metformin over 14 years, with a dose of 1,000 mg BID at the time of admission. He had good diabetic control with a well-balanced diet and a hemoglobin (Hgb) A1C of 7.0% upon admission. Other notable medications include apixaban 5 mg BID and a daily 81 mg dose of acetylsalicylic acid. The patient presented to the hospital after suffering a fall onto the stone border of his fireplace, which caused a large lesion on his right thigh. He stated that he had been suffering from frequent falls over the past six months and that he had tried physical therapy without any resolution of symptoms. The patient believed that his dizziness and unsteadiness were progressive and steadily worsening over the years. He described the need to frequently reposition himself to prevent himself from falling, stating that he suffered from multiple falls over the past few years and that he required a cane at one point in time. The patient denied any recent weakness, palpitations, chest pain, or loss of consciousness leading to his falls. However, his family did note some mild forgetfulness of deadlines and appointments over the previous months.

**Figure 1:**
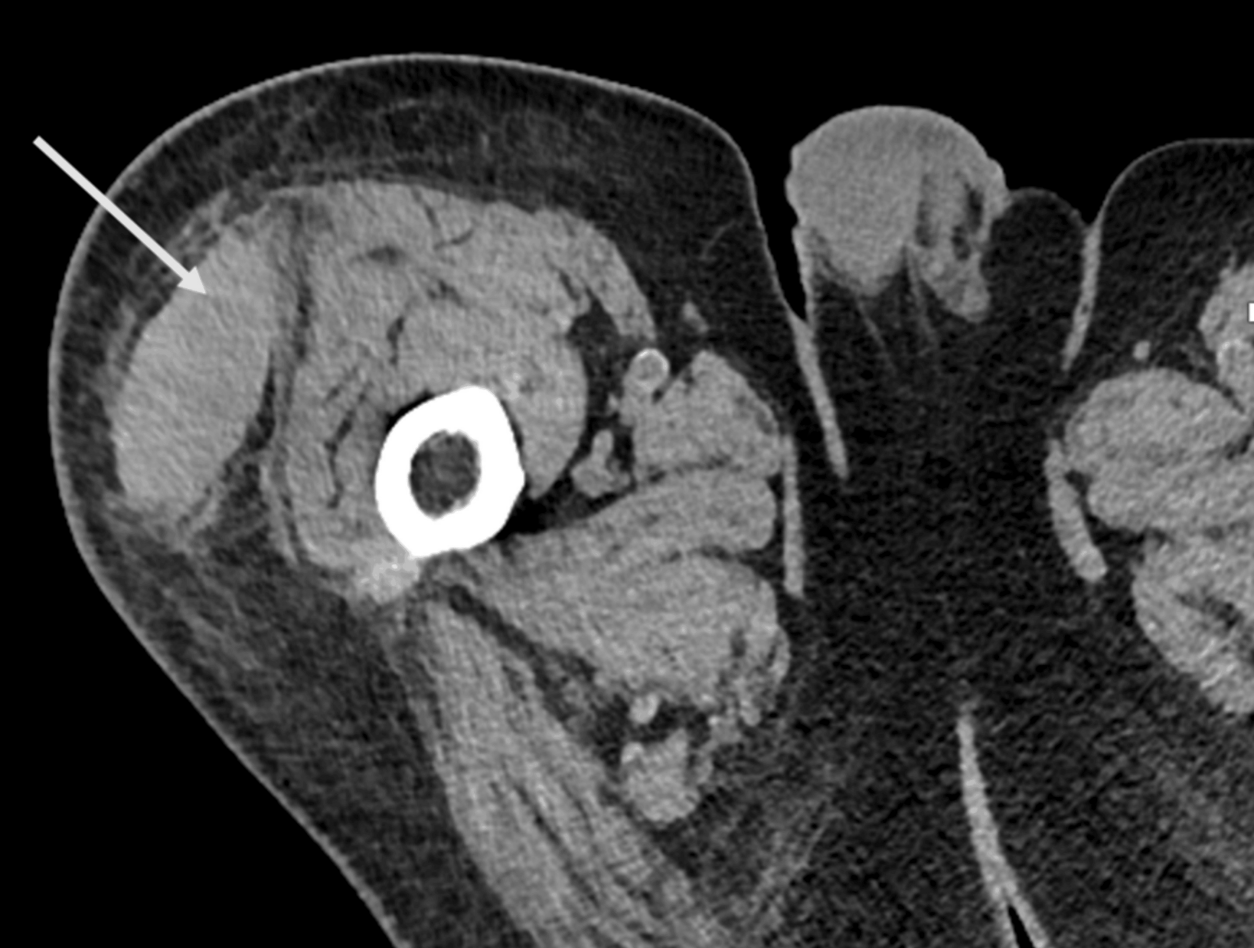
Noncontrast CT-Cross-Sectional View of the Right Thigh CT image revealing a deep subcutaneous fluid collection measuring 6.6 x 3.5 cm, with the arrow at the interface of the deep fascia and adjacent tensor fascia lata muscle. Overall, this lesion is consistent with an MLL due to the location of the lesion being between the fascia and overlying subcutaneous fat.

On admission, his vitals were stable, and a physical exam revealed a large ecchymosis with associated swelling along the right hip and right anterior and lateral thigh. These areas were firm and tender, but compressible with strength and sensation intact. On neurological exam, the patient was alert and oriented with 5/5 strength throughout. However, he did exhibit diminished lower extremity vibratory sensation along with truncal ataxia while sitting and standing. The patient’s imaging included a CT scan, which was notable for a relatively high-density deep subcutaneous fluid collection at the interface of the deep fascia and adjacent tensor fascia lata measuring 6.6 x 3.5 cm with no evidence of fracture (Figure 1). The overall impression of the CT scan suggested an MLL due to the location of the lesion being between the fascia and overlying subcutaneous fat, differentiating it from a simple hematoma, which would not typically accumulate in this potential space. The patient’s laboratory results are presented in Table 1. Further testing revealed negative intrinsic factor antibodies and antiparietal cell antibodies.

**Table 1:** Laboratory Results on Admission Labs are notable for decreased hemoglobin (Hgb), hematocrit (Hct), and vitamin B12 levels and increased homocysteine and methylmalonic acid (MMA) levels.

Lab parameter	Admission value	Reference range
Hemoglobin (Hgb)	10.2 g/dL	14-18 g/dL
Hematocrit (Hct)	32.6%	40%-54%
Mean corpuscular volume (MCV)	93.1 fL	82-98 fL
Platelets	250,000/mL	150,000-350,000/mL
Serum iron	62 mcg/dL	49-181 mcg/dL
Ferritin	106 ng/mL	10-385 ng/mL
Total iron-binding capacity (TIBC)	274 mcg/dL	250-450 mcg/dL
Transferrin saturation (TSAT)	22.6%	20%-50%
Folate	9.60 ng/mL	2-17 ng/mL
Vitamin B12	156 pg/mL	200-900 pg/mL
Homocysteine	13.9 mmol/L	Normal < 11.4 mmol/L
Methylmalonic acid (MMA)	379 nmol/L	87-318 nmol/L

The patient’s hospital course involved a consult to general surgery for his leg lesion, which rapidly improved without any intervention other than a gentle compression wrap. The patient also received an intramuscular B12 injection on hospital day two, and by his discharge on hospital day seven, his Hgb rose to 11.1 g/dL and hematocrit (Hct) rose to 36.2%.

Since his discharge, the patient has received monthly B12 injections with his most recent B12 level being 603 pg/mL and his Hgb and Hct within normal limits. The patient also reported much-improved balance and less frequent falls since his discharge.

## Discussion

This is the first reported case of an MLL occurring in a patient with a B12 deficiency secondary to long-term metformin use. There have been reported cases of vitamin B12 deficiency that arise with the use of metformin, and this has prompted further studies on the possible mechanism of this interaction and the prevalence of this condition. Various studies have found that 7%-32% of patients on metformin therapy developed a vitamin B12 deficiency [4-7]. Higher dosages of metformin (greater than 1,000 mg) and treatment duration greater than four years have also been found to significantly increase the risk of B12 deficiency [6].

Metformin use leads to vitamin B12 deficiency primarily through interference with calcium-dependent absorption of the B12-intrinsic factor complex in the terminal ileum [8]. Furthermore, there has been a high prevalence of B12 deficiency among diabetic patients treated with metformin showing that there is an immune-metabolic mediating effect in this process [7]. This induced deficiency can be treated with monthly B12 injections as was the case for our patient. One study has found that calcium supplementation could also reverse the metformin-associated vitamin B12 malabsorption due to the dependence of calcium on the receptors' uptake of B12 in the terminal ileum [8].

Our patient’s history suggests longstanding B12 deficiency with associated proprioceptive deficits resulting in falls, perhaps over a period of years. Notably, he had symptoms consistent with B12 deficiency including neuropathy and ataxia without the presence of macrocytic anemia. In the absence of antibodies associated with pernicious anemia, his metformin is almost surely the cause of his B12 deficiency. A similar report of an 82-year-old patient on long-term metformin with proprioceptive deficits that resolved after supplementing vitamin B12 has been reported [9].

Deficiency of vitamin B12 leads to central nervous system (CNS) dysfunction and proprioceptive deficits through a variety of mechanisms. First and perhaps most importantly, vitamin B12 is essential for maintaining the integrity of myelin, a protective sheath surrounding nerves. Without adequate amounts of vitamin B12, demyelination can occur especially in the lateral and posterior columns of the spinal cord, which regulate motor and proprioception and vibration, respectively [10]. When this demyelination is severe enough, it can also lead to subacute combined degeneration. Additionally, vitamin B12 deficiency can further lead to myelin damage by increasing levels of neurotoxic cytokines such as tumor necrosis factor-alpha (TNF-alpha) and decreasing neurotrophic cytokines such as interleukin-6 (IL-6) [11]. The imbalance of these factors further leads to increased myelin damage and neuronal dysfunction. Due to the combination of these deleterious effects, a frequent complication of vitamin B12 deficiency is balance and proprioception issues, which our patient suffered from for years preceding his most recent fall leading to his admission.

Our case is unique in that our patient developed an MLL after his fall. These lesions usually only occur from high-energy trauma or shearing forces often presenting with underlying fractures, indicating that there may have been other factors leading to the development of his MLL along with his moderate-impact fall on his stone fireplace, especially since there was not an underlying fracture. The underlying pathophysiology of the development of an MLL involves shearing forces creating a cavity in between fascial layers and subcutaneous tissues [3]. These shearing forces lead to the disruption of lymphatic vessels and capillaries in these newly formed cavities, leading to the accumulation of hemolymphatic fluid and a subsequent inflammatory cascade eventually culminating in an MLL. If left undiagnosed or untreated, MLLs can lead to dangerous complications including skin necrosis and deep wound infections [12]. Our patient used an anticoagulant and antiplatelet medication, which further contributed to his bleeding and likely played a part in the development of his lesion. However, this alone likely would not have been enough to lead to the development of his MLL.

While there have been no clear associations between vitamin B12 deficiencies leading directly to MLLs, there have been studies that illustrate how vitamin B12 impacts bleeding, clotting, and wound repair. Vitamin B12 deficiency can lead to thrombocytopenia and platelet dysfunction [13,14]. While our patient had normal platelet levels, impaired platelet aggregation has been observed in patients with B12 deficiency, and this could have been one contributing factor to our patient’s MLL.

Vitamin B12 is also integral for DNA synthesis and cellular repair, which is crucial for wound healing. Deficiencies of B12 lead to increased levels of homocysteine, which is toxic to endothelial cells and can impair the integrity of blood vessels by reducing blood flow to the site of tissue injury. Increased homocysteine levels have also been associated with increased reactive oxygen species and inflammatory cytokines, impaired collagen synthesis, and reduced angiogenesis contributing to chronic venous ulcers and poor wound healing [15-17]. Therefore, there is a strong possibility that the elevated homocysteine levels from our patient’s B12 deficiency contributed to reduced vascular integrity and poor wound healing, and this combined with impaired platelet aggregation and a fall secondary to proprioceptive deficits from B12 deficiency led to the formation of the MLL in our patient. Further studies will need to be conducted on patients with MLLs to further elucidate all potential contributing factors to these rare lesions.

## Conclusions

Our case illustrates how long-standing metformin use can lead to vitamin B12 deficiency, which may cause profound neurological symptoms contributing to falls and injuries, especially in elderly patients. Our patient developed an MLL after a moderate-impact fall due to metformin-induced B12 deficiency, with his anticoagulation and worsened wound healing from B12 deficiency having potentially contributed to the severity of his lesion. Although we were able to treat our patient’s B12 deficiency and his MLL with conservative measures, his case serves as a reminder of the potentially serious complications that can arise due to B12 deficiencies. In the future, it will be important for clinicians to be aware of metformin-induced vitamin B12 deficiencies to avoid associated complications for their patients and to initiate early screening and treatment.
